# A Systematic RNAi Screen Reveals a Novel Role of a Spindle Assembly Checkpoint Protein BuGZ in Synaptic Transmission in *C. elegans*

**DOI:** 10.3389/fnmol.2017.00141

**Published:** 2017-05-11

**Authors:** Mei Han, Wenjuan Zou, Hao Chang, Yong Yu, Haining Zhang, Shitian Li, Hankui Cheng, Guifeng Wei, Yan Chen, Valerie Reinke, Tao Xu, Lijun Kang

**Affiliations:** ^1^Key Laboratory of Medical Neurobiology of the Ministry of Health of China, Department of Neurobiology, Institute of Neuroscience, Zhejiang University School of MedicineHangzhou, China; ^2^National Laboratory of Biomacromolecules, CAS Center for Excellence in Biomacromolecules, Institute of Biophysics, Chinese Academy of SciencesBeijing, China; ^3^Department of Genetics, Yale University School of MedicineNew Haven, CT, USA

**Keywords:** RNAi screen, synaptic transmission, *C. elegans*, C2H2 zinc-finger protein, synaptic vesicles

## Abstract

Synaptic vesicles (SV) store various neurotransmitters that are released at the synapse. The molecular mechanisms of biogenesis, exocytosis, and endocytosis for SV, however, remain largely elusive. In this study, using Complex Object Parametric Analysis and Sorter (COPAS) to monitor the fluorescence of synapto-pHluorin (SpH), we performed a whole-genome RNAi screen in *C. elegans* to identify novel genetic modulators in SV cycling. One hundred seventy six genes that up-regulating SpH fluorescence and 96 genes that down-regulating SpH fluorescence were identified after multi-round screen. Among these genes, *B0035.1 (bugz-1)* encodes ortholog of mammalian C2H2 zinc-finger protein BuGZ/ZNF207, which is a spindle assembly checkpoint protein essential for mitosis in human cells. Combining electrophysiology, imaging and behavioral assays, we reveal that depletion of BuGZ-1 results in defects in locomotion. We further demonstrate that BuGZ-1 promotes SV recycling by regulating the expression levels of endocytosis-related genes such as rab11.1. Therefore, we have identified a bunch of potential genetic modulators in SV cycling, and revealed an unexpected role of BuGZ-1 in regulating synaptic transmission.

## Introduction

Synaptic vesicles (SV) store neurotransmitters, concentrate in the presynaptic nerve terminals, and undergo Ca^2+^-dependent exocytosis. These steps include biogenesis of SVs, transport to release sites, docking with plasma membrane, priming, and calcium-triggered fusion (Sudhof and Rizo, 2011; Rizo and Xu, 2015). After exocytosis, SVs undergo endocytosis, recycle, and refilling with neurotransmitters for next round of exocytosis (Wu et al., 2014; Rizo and Xu, 2015; Xie et al., 2017). Three modes of exocytosis, including full-collapse fusion, kiss-and-run, and compound exocytosis, are coupled to classical endocytosis, kiss-and-run, and bulk endocytosis, respectively (Wu et al., 2014; Rizo and Xu, 2015; Xie et al., 2017).

The fusion of SVs to the plasma membrane is mechanically driven by the interaction among SNARE complex, a four-helix coiled-coil structure, which consists of a vesicle SNARE (v-SNARE) protein, synaptobrevin (on vesicle membrane), and two target membrane SNARE (t-SNARE) proteins, syntaxin-1, and SNAP-25 (on the plasma membrane) (Sudhof and Rizo, 2011; Südhof, 2013; Rizo and Xu, 2015). Fusion-competent conformations of SNARE proteins are maintained by chaperone complexes including CSPα, Hsc70, and SGT (Sudhof and Rizo, 2011). The synaptic SNARE and SM fusion-machine is controlled by synaptotagmin by Ca^2+^ via synaptotagmin and complexin, and is additionally regulated by a presynaptic active zone proteins that includes Munc13 and RIM as central components (Sudhof and Rizo, 2011). Classical endocytosis is clathrin-dependent (Wu et al., 2014). All three SNARE proteins that catalyze exocytosis—synaptobrevin, SNAP25, and syntaxin are also needed for endocytosis initiation (Sudhof and Rizo, 2011; Wu et al., 2014; Xie et al., 2017). Some molecules such as amphiphysin, endophilin, AP180, auxilin, and dynamin have been implicated to be involved in endocytosis (Wu et al., 2014). Additionally, endocytosis is prolonged by depletion of clathrin, AP2, stonin 2, endophilin, and auxilin (Wu et al., 2014). Although the principle steps and some molecules have been identified, the exact mechanisms of biogenesis, exocytosis and endocytosis of SVs remain largely elusive.

Synaptobrevin is the key molecule on SVs, so synapto-pHluorin (SpH), a pH-sensitive variant of GFP (pHluorin) fused to the luminal domain of synaptobrevin, is widely used to quantitatively measure the exocytosis and endocytosis of SVs (Miesenböck et al., 1998; Sankaranarayanan et al., 2000; Dittman and Kaplan, 2006; Afuwape and Kavalali, 2016). At rest, SpH fluorescence is quenched by the luminal acidic pH of the vesicle. After stimulation, vesicles fuse with the plasma membrane exposing the lumen to the neutral pH of the extracellular medium and causing an increase in SpH fluorescence. The fluorescence is then quenched once again after endocytosis and reacidification (Sankaranarayanan et al., 2000; Afuwape and Kavalali, 2016).

*C. elegans* is an excellent model system for studying SV cycling and performing systematic RNAi screen (Richmond and Broadie, 2002; Jadiya et al., 2016). With the availability of the *C. elegans* whole-genome RNAi feeding library, the expression of endogenous genes can be specifically knocked down by feeding bacteria expressing double-stranded RNA (dsRNA) of corresponding genes (Kamath et al., 2003). In order to get a clearer picture of the SV cycling, we aimed to identify novel genes required for synaptic vesicle cycling via *C. elegans* whole-genome RNAi screen, using SpH as the probe. Previous whole genome RNAi screens in *C. elegans* mostly detected and scored phenotypes by eyes, with limited quantifiable results, quality control, and systematic analysis. In this study, we detected the SpH fluorescence intensity of individual worms via Complex Object Parametric Analysis and Sorter (COPAS, Union Biometrica), which provides a method for high-throughput, reproducible quantitative analysis (Pulak, 2006; Dupuy et al., 2007). We screened two RNAi feeding libraries, including both Ahringer Library and Vidal Library (Kamath et al., 2003; Rual et al., 2004; Kim et al., 2005), together covering 94% of predicted *C. elegans* genes. We identified 176 genes up-regulating SpH fluorescence and 96 genes down-regulating SpH fluorescence after multi-round screen. Among these candidate genetic modulators of SV cycling, the C2H2 zinc-finger protein BUGZ-1, an ortholog of mammalian spindle assembly checkpoint protein BuGZ/ZNF207, is critically required for SV cycling, suggesting an unidentified role of spindle assembly checkpoint proteins in synaptic transmission.

## Results

### A whole-genome RNAi screen identifies novel genes required for synaptic vesicle cycling

To identify novel genes related to synaptic vesicle (SV) cycling, we performed an automatic whole-genome RNAi screen in *C. elegans*. We used a pan-neuronal expressed *snb-1* promoter to drive *SNB-1::pHluorin* (SpH) expression in the nerve system for optical measurements of presynaptic activity. SpH is a PH-sensitive variant of GFP fused to the luminal domain of synaptobrevin. Previous studies have confirmed that the fluorescence of SpH is quenched in the acidic environment of the SV lumen but increased dramatically when the SVs fused to the plasma membrane (Miesenböck et al., 1998; Sankaranarayanan et al., 2000; Dittman and Kaplan, 2006; Afuwape and Kavalali, 2016). The transgenic strain *Is[snb-1::pHluorin]* was crossed with the RNAi hypersensitive strain KP3948 *eri-1(mg366); lin-15b(n744)* (Sieburth et al., 2005) to generate worm for whole-genome RNAi screen. We performed screen with COPAS, a machine which is able to effectively detect the changes of fluorescence intensity in *C. elegans* by line scanning, and profile the fluorescent intensity for each worm (Figure S1, Dupuy et al., 2007; Han et al., 2013). For high-throughput screen using COPAS, worms were incubated in liquid culture in standard flat-bottomed 96-well plates according to previous reports with some modification (Lehner et al., 2006). We developed series of programs to batch process the raw data of COPAS. Relative florescent signals (RFS) were used to represent the SpH fluorescent signal, and robust Z-score were calculated to normalize SpH fluorescent intensity from different experimental 96-well plates (see Materials and Methods).

We chose *unc-11/AP180*, which has been reported to play an important role in recycling synaptobrevin from plasma membrane (Nonet et al., 1999; Dittman and Kaplan, 2006; Sudhof and Rizo, 2011), as an up-regulated control (up regulate SpH fluorescent intensity). Knocking down *unc-11* AP180 6-fold increased the fluorescent intensity of SpH in the probe strain (Figure S1). We used the RNAi bacteria expressing dsRNA of *gfp*, whose sequence is similar to pHluorin, as a down-regulated control (down regulate SpH fluorescent intensity). Knocking down pHluorin by feeding worms with *gfp* RNAi bacteria decreased the fluorescent intensity of SpH to <0.5-fold in worms. The fluorescent intensities of worms cultured in 96-well plates in liquid which detected by COPAS were consistent with the fluorescent intensities of worms cultured on RNAi plates which detected by the confocal microscope. These results indicated that positive genes can be knocked down by feeding worms with corresponding RNAi bacteria in liquid culture, and the changes of fluorescent intensity can be detected efficiently by the COPAS.

**Figure 1:**
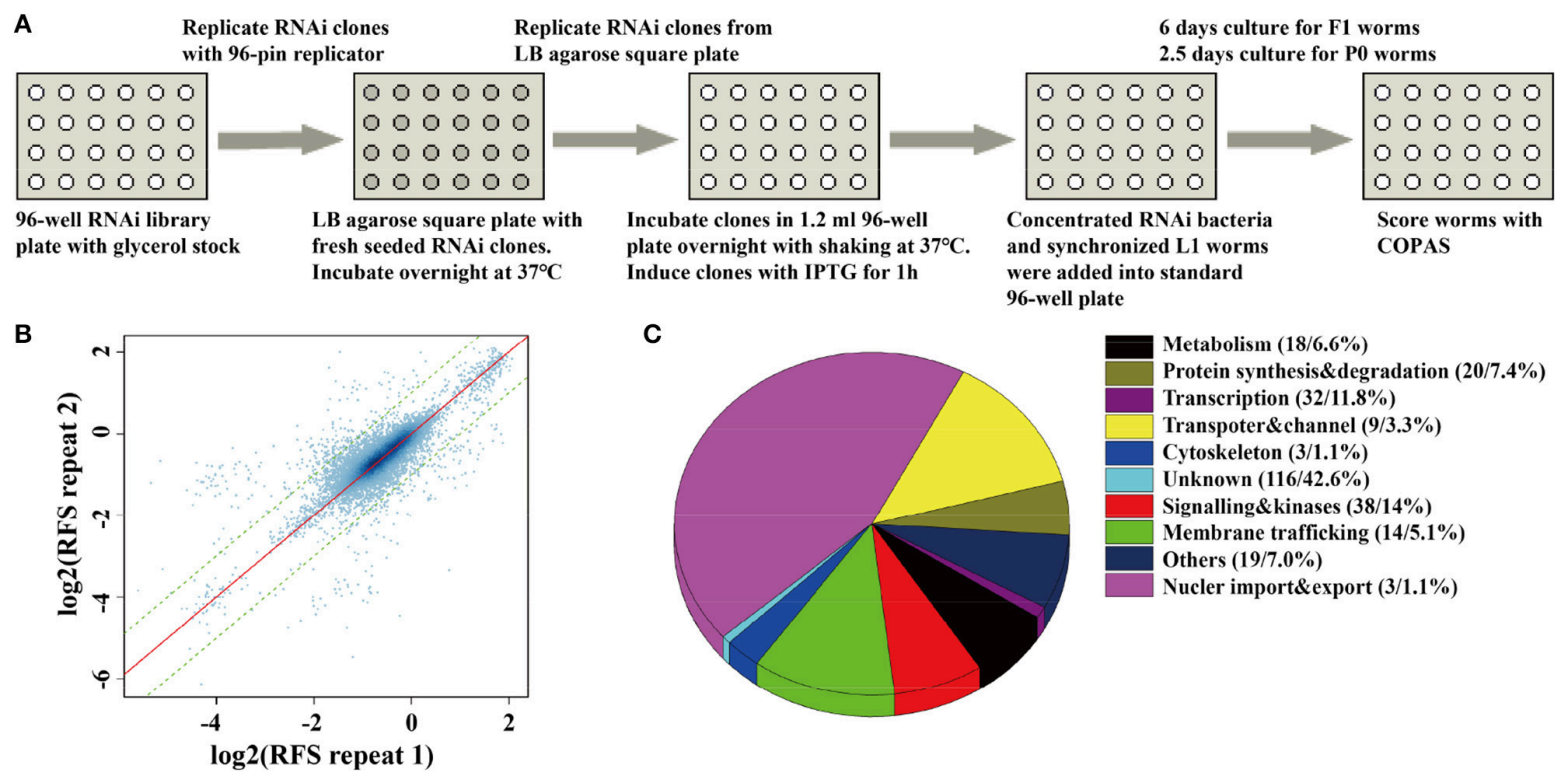
**A whole-genome RNAi screen for genes required for synaptic vesicle cycling**. **(A)** A schematic diagram of COPAS-based whole-genome RNAi screen. RNAi experiments for each clone were duplicated for whole genome screen or four replicates for the second validation screen. **(B)** The reproducibility of the whole genome screen data. The scatter plot shows the reproducibility of two repeats of each RNAi experiment in the whole genome screen. Each point indicates a RNAi experiment. The x axis and y axis are the log2 transformed median RFS (relative fluorescent signal) of the two repeats. The red line y = x indicates 100% repeatability. The two green lines y = x+1 and y = x−1 are the fold change of the two repeats are equal to 2. 97.9% of the points fall between the two lines. **(C)** The percentage distribution chart of candidate genes within indicated functional classes. The total number of genes in each group and its percent to the total number of candidate genes are indicated in the parenthesis. 272 candidate genes were found for synaptic vesicle cycling after multi-round RNAi screen (Tables S2, S3).

In order to monitor the quality of RNAi treatment for each 96-well plate, we added up-regulated control of *unc-11* RNAi clones, down-regulated control of *gfp* RNAi clones, and empty vector control of L4440 clones into the empty wells of each 96-well plate. We rearranged a few clones in 96-well plates to insure each experimental plate contains two empty vector controls, one up-regulated control and one down-regulated control. Synchronized L1 worms were fed with each RNAi clone in each well of 96-well plates, and SpH fluorescence was detected in their progeny via the COPAS. For those RNAi clones that caused embryonic lethal or sterile, SpH fluorescence was detected in young adults of the same generation (Figure 1A). All bacteria RNAi clones were duplicated in the whole-genome screen. The plates with low repeatability (fold change of the florescent signals between the two repeats larger than 1.5) were redone. We examined the repeatability of two repeats for each clone, the overall correlation of the paired repeats is 0.81, which means that most paired repeats have very similar values (Figure 1B). Based on the genomic distribution of fluorescent intensity change after RNAi treatment, genes fell in the two tails (5%) were chosen as candidates. The candidates were rearranged into new 96-well plates with controls for further validation. In the secondary validation, worms were retested under the same condition. Each RNAi clones had four repeats. Genes that caused stable and consistent SpH signal changes were chosen as positive hits.

The fluorescent intensity changes of SpH could also due to unspecific reasons, especially the changing of protein expression level, so it is necessary to exclude unrelated genes for SV cycle. We checked the expression level of GFP in neurons, using the RNAi hypersensitive worm strain *nre-1(hd20);lin-15b(hd126);rhIs13[unc-119p::GFP* + *dpy-20(*+*)]* (Schmitz et al., 2007). Genes that lead to significant fluorescent intensity changes (*p* < 0.05) were excluded. Finally, 176 genes with up-regulated fluorescent intensity of SpH and 96 genes with down-regulated fluorescent intensity of SpH were identified after multi-round screen (Table S2). Seventy-six percent of these genes are evolutionarily conserved. The functional classes of candidate genes indicated that diverse groups of genes were taken part in SV cycling (Figure 1C and Table S3). Although some genes were already identified to be required for SV cycling, there still a big portion of genes with less known, which maybe new important regulators for SV cycling.

### Mutants of candidate genes display acetylcholine release defects

**Figure 2:**
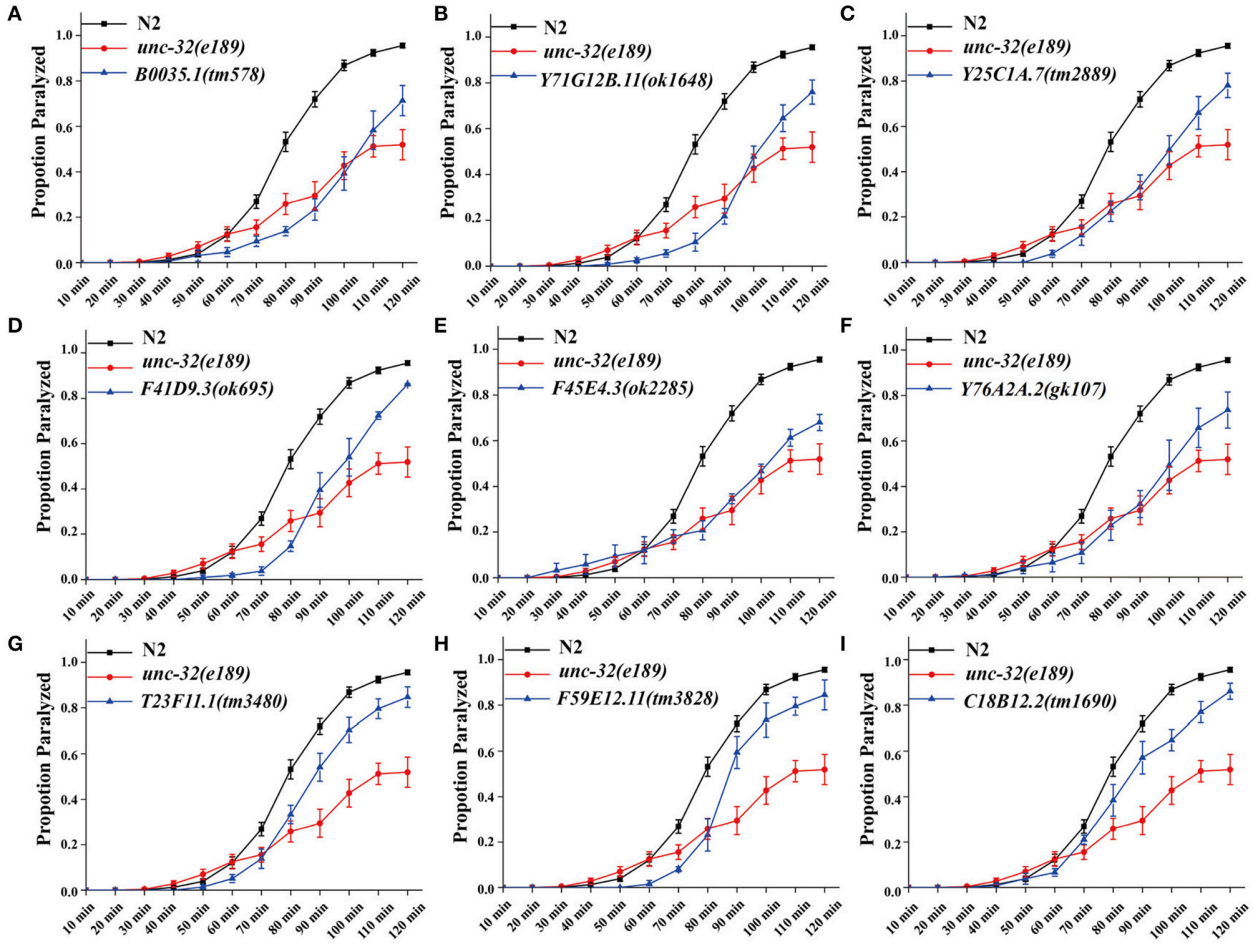
**Deletion mutants of candidate genes show acetylcholine secretion defects**. Acetylcholine release was detected by determining the proportion of paralyzed animals exposed to 1 mM aldicarb. **(A–I)** Proportion paralyzed over time from indicated deletion mutant worms. Worm strains of *B0035.1(tm578), Y71G12B.11(ok1648), Y25C1A.7(tm2889), F41D9.3(ok695), F45E4.3(ok2285), Y76A2A.2(gk107), T23F11.1(tm3480), F59E12.11(tm3828)*, and *C18B12.2(tm1690)* exhibited aldicarb-resistance. *UNC-32(e189)* as aldicarb resistant positive control. 25–35 young adult animals were tested for each experiment, at least three independent experiments were performed. Error bars represent SEM.

In *C. elegans*, steady-state acetylcholine (ACh) secretion can be indirectly detected by measuring their resistance to the acetylcholine esterase inhibitor aldicarb (Mahoney et al., 2006b). Accumulation of ACh at synapsis caused by aldicarb leads to acute paralysis of worms and finally death. Blocking of SV cycle could lead to less release of ACh, which can be detected by aldicarb resistance analysis (Lackner et al., 1999; Mahoney et al., 2006b). To validate the participation of candidates in neurotransmitter release, we examined the resistance to aldicarb in 14 mutations which lead to dramatic SpH fluorescent intensity changes when knocking down by RNAi treatment. We used *unc-32(e189)* as a positive control in aldicarb resistance assay (Wiese et al., 2012). *unc-32* encodes an ortholog of subunit of the membrane-bound domain of vacuolar proton-translocating ATPase. *unc-32(e189)* is a specific mutant that is important for locomotion and SV morphology in motoneurons (Wiese et al., 2012). Nine mutants showed significant aldicarb-resistance phenotypes, including *B0035.1(tm578), Y71G12B.11(ok1648), Y25C1A.7(tm2889), F41D9.3(ok695), F45E4.3(ok2285), Y76A2A.2(gk107), T23F11.1(tm3480), F59E12.11(tm3828), C18B12.2(tm1690)* (Figure 2A). The other five mutants showed no resistance (data not shown). All these candidate genes we identified are evolutionarily conserved, of which orthologs can be found in human, mouse, and fly, indicating their critical roles in diverse species.

In order to identify whether candidate genes function in presynaptic or postsynaptic terminal, we examined their resistance to the drug levamisole. Levamisole is a cholinergic receptor agonist that directly activates postsynaptic ACh receptors (Richmond and Jorgensen, 1999; Culetto et al., 2004). Wild type worms and mutants defected in presynaptic terminal display similar paralyzed phenotypes. All these 9 mutants exhibited similar paralysis rates with wild type (Figure S2), indicating a presynaptic function of candidate genes. Besides, SpH fluorescent intensities were significantly changed when knocking down these genes with RNAi treatment in the primary and the secondary screen, while GFP signals unaltered in nerve system of VH624 worms after RNAi treatment. These results also indicated that these genes probably function in presynaptic nerve terminal.

### BuGZ-1 is a C2H2 zinc-finger protein expressed in the nuclei of neurons and muscles

**Figure 3:**
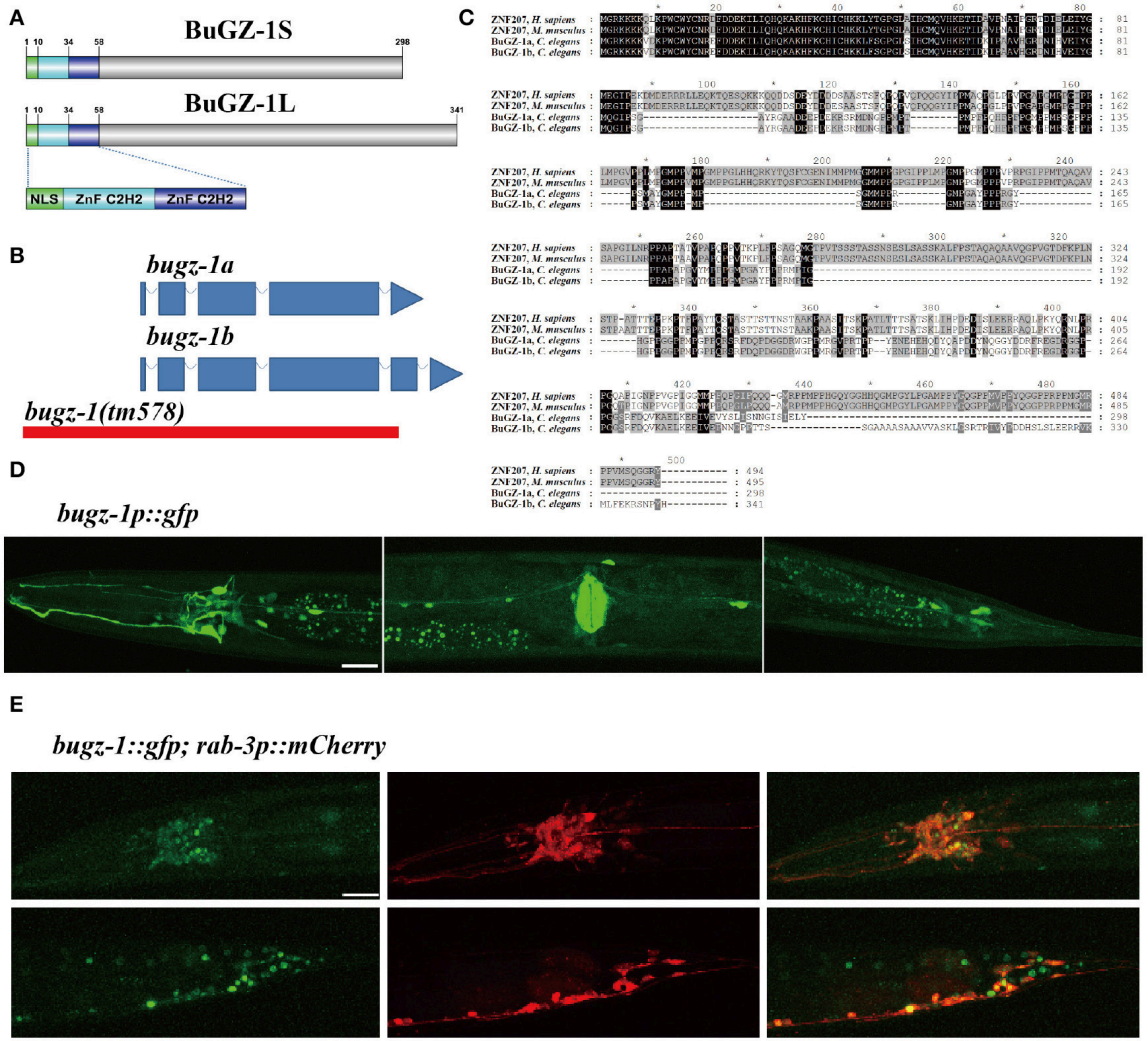
**BuGZ-1 is an evolutionary conserved protein with a predicted function as transcription factor and is mainly expressed in the nervous system. (A)** Schematic diagram of protein domain structure of two isoforms of BuGZ-1. Both short isoform BuGZ-1S and long isoform BuGZ-1L have a nuclear localization signal (NLS) motif and two zinc finger C2H2 (ZnF C2H2) domains. **(B)** Schematic diagram of gene structure for *bugz-1a* and *bugz-1b*. Blue boxes indicate exons; blue lines indicate introns. 8X backcross of *bugz-1(tm578)* to wild type was performed before doing experiments. **(C)** Multiple sequence alignment of the highly conserved N-terminal of BuGZ-1 from different species, including humans (*H. sapiens*), mouse (*M. musculus*), and worm (*C. elegans*). Perfect sequence conservation is indicated in black. **(D)** Transcriptional expression pattern of *bugz-1p::gfp. bugz-1* is expressed in the nervous system, intestine and muscles. Images were taken from head, valve, and tail in ventral view, head to the left. **(E)** Translational expression pattern of *bugz-1::gfp*. Neurons are marked with *rab-3p::mCherry*. Images were taken from head and tail in lateral view, head to the left. BuGZ-1 is mainly localized to the nuclei of neurons. Scale bars indicate 20 μm.

Among these 9 candidate genes, *B0035.1 (bugz-1)* encodes an ortholog of mammalian C2H2 zinc-finger protein BuGZ/ZNF207. Very interestingly, recent studies have been implicated that mammalian BuGZ binds to and stabilizes spindle check point protein Bub3 during interphase and facilitates mitosis (Jiang et al., 2014; Toledo et al., 2014). Depletion of BuGZ in cancer cells causes chromosome misalignment and mitotic arrest followed by massive cell death (Jiang et al., 2014; Toledo et al., 2014). Our observation implicates that BUGZ may also have an unidentified role in synaptic transmission, thus we focused our following study on BUGZ-1. The *C. elegans* gene *bugz-1* encodes two isoforms of BuGZ-1, BuGZ-1S (B0035.1a) and BuGZ-1L (B0035.1b), both containing a nuclear localization sequence (NLS) and two C2H2 zinc finger domains at the N-terminal of the protein (Figures 3A,B). BUGZ-1 is highly conserved from worm to human and exhibits 68% identity between amino acids 1 and 107 of mouse and human homologs (Figure 3C). We generated a *bugz-1p::gfp* construct by driven GFP under the promoter of the *bugz-1* gene. GFP signals were observed throughout nervous system and muscles, including the nerve ring and ventral nerve cord (Figure 3D). In order to confirm the nervous system expression of BuGZ-1, we co-injected the constructs of *bugz-1p::gfp* and *rab-3p::mCherry*. BUGZ-1 is co-localized well with RAB-3 in most neurons in the nerve ring and the tail. Notably, BUGZ-1 is localized to the nuclei of these cells, consistent with the subcellular location of mammalian BuGZ (Figure 3E, Jiang et al., 2014; Toledo et al., 2014).

### Depletion of BuGZ-1 results in locomotion defects

**Figure 4:**
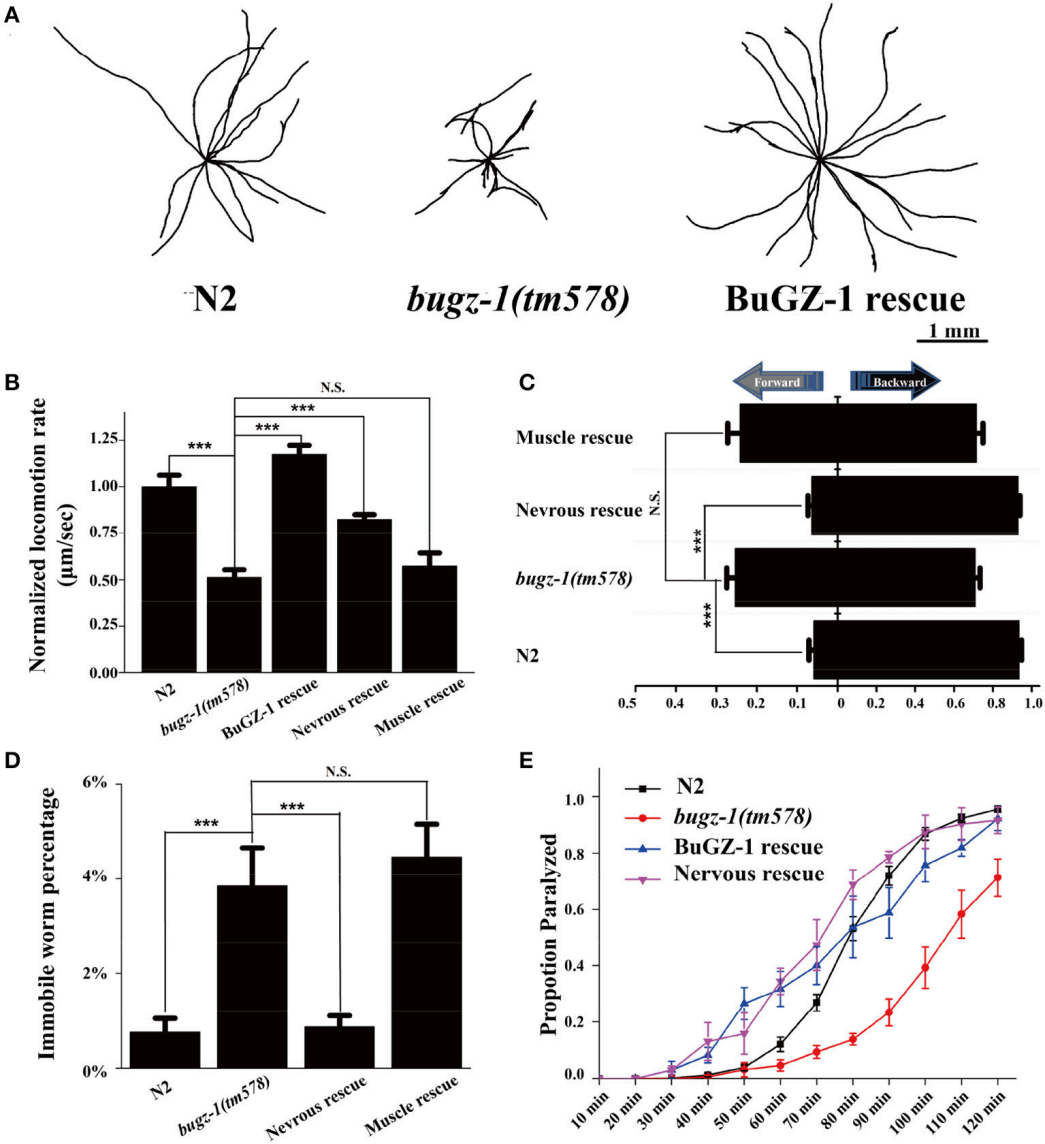
**BuGZ-1 regulates acetylcholine secretion and worm locomotion. (A,B)** *bugz-1(tm578)* animals displayed more hesitating and uncoordinating movement with decreased trajectories **(A)** and locomotion speed **(B)**. The locomotion rate is the average velocity of each worm in 40 seconds. **(C,D)** *bugz-1(tm578)* mutant worms prefer to move backward or stay immobile. **(C)** Statistic comparison of average proportion of time spent on moving forward or backward for indicated worms. **(D)** Percentage of worms stay immobile in indicated genotypes. All the locomotion defects of *bugz-1(tm578)* can be rescued by endogenous expressed BuGZ-1 (*bugz-1::gfp*) and nervous expressed BuGZ-1 (*rab-3p::bugz-1::mCherry*) but not muscle expressed BuGZ-1 (*myo-3p::bugz-1::mCherry*) in mutation background. The number of worms analyzed for locomotion is *n* = 15 for each genotype. **(E)** Proportion paralyzed over time from indicated worm strains exposed to 1 mM aldicarb. Aldicard resistance defect of *bugz-1(tm578)* was rescued by endogenous expressed BuGZ-1 and nervous expressed BuGZ-1 but not muscle expressed BuGZ-1 in mutation background. 25–35 young adult animals were tested for aldicarb resistant experiment, at least three independent experiments were performed. Values that significantly differ from controls are indicated (^***^p < 0.001 by two-tails Student's *t*-test). N.S. indicates no significant difference from worm genotypes compared. Error bars represent SEM.

To further investigate the function of BuGZ-1 in synaptic vesicle cycling, we used a mutant strain *bugz-1(tm578)* generated by the National Bioresource Project (Tokyo, Japan). Five out of six exons are deleted in *bugz-1(tm578)* mutant worms. The deletion starts from the promoter region of *bugz-1*, which makes *bugz-1(tm578)* a null allele (Figure 3B). While wild type animals exhibited a smooth and continuous sinusoidal movement, *bugz-1(tm578)* animals displayed more hesitating and uncoordinating movement with decreased trajectories and locomotion speed (Figures 4A,B). Moreover, mutants prefer to move backward (ratio of total time = 25.3 ± 10.4%) or stay immobile (3.85 ± 4.01%) when compared with wild type worms (5.8 ± 7.3% and 0.76 ± 1.66%, respectively) (Figures 4C,D).

### BuGZ-1 regulates neurotransmitter release at NMJs

*C. elegans* locomotion is controlled by nervous system that innervate specific muscles. Defects of either neuronal or muscle activities lead to locomotion abnormal (Richmond and Jorgensen, 1999; Dittman and Kaplan, 2006). We observed that *bugz-1* mutants showed a dramatic resistance to aldicarb, an inhibitor for acetylcholine esterase (Dittman and Kaplan, 2006; Mahoney et al., 2006b, Figure 4E). The aldicarb-resistance phenotype and locomotion defects in *bugz-1* mutants can be rescued by transgenic expression of BUGZ-1 cDNA driven by its own promoter or by the pan-neuronal promoter *rab-3* (Figures 4B–E, Mahoney et al., 2006a), suggesting that BuGZ-1 predominately functions in nervous system to regulate synaptic transmission.

**Figure 5:**
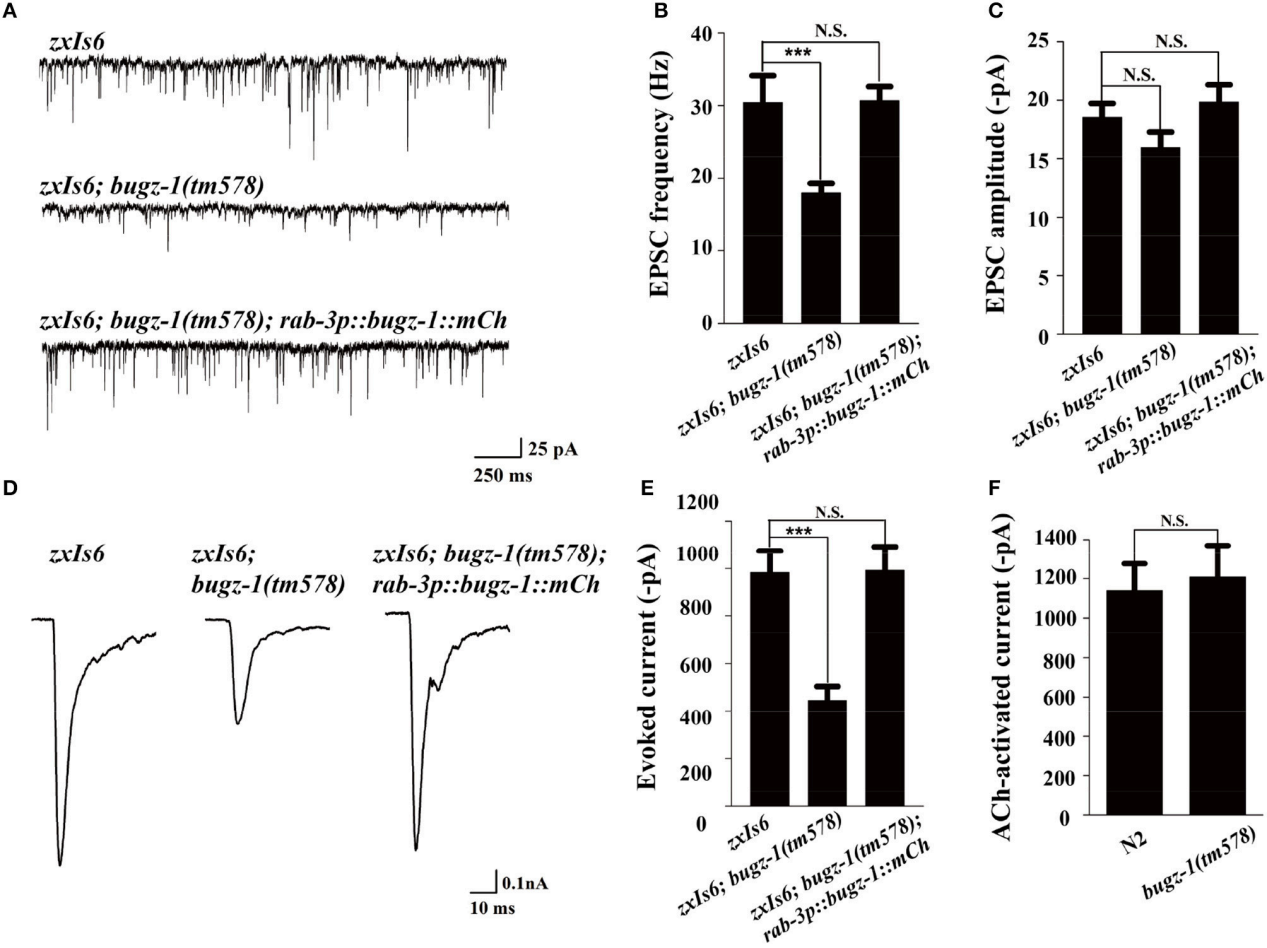
**BuGZ-1 regulates neurotransmitter release at NMJs**. **(A–C)** Recording of endogenous excitatory postsynaptic currents (EPSC) from adult body wall muscles of the indicated genotypes. *zxIs6 [unc-17p::chop-2(H134R)::yfp, lin-15(*+*)]*. Representative traces **(A)** and summary data for endogenous EPSC frequency **(B)** and EPSC amplitude **(C)** are shown. *n* ≥ 15. **(D,E)** Recording of photo-evoked EPSCs from adult body wall muscles of the indicated genotypes. ChR2-expressing cholinergic motor neurons were photo-activated by 100 ms blue light. Representative traces **(D)** and summary data **(E)** are shown. *n* ≥ 10. **(F)** Acetylcholine-induced currents in body muscles of mutant were indistinguishable from wild type. *n* ≥ 10. For each genotype, independent experiments were carried out in different animals. Values that significantly differ from controls are indicated (^***^p < 0.001 by two-tails Student's *t*-test). N.S. indicates no significant difference from control. Error bars represent SEM.

We then directly measured neurotransmitters release by recording the endogenous excitatory postsynaptic currents (EPSC) and evoked EPSCs at NMJs in *zxIs6* animals, in which a light-gated cation channel, channelrhodopsin-2 (ChR2), is expressed specifically in cholinergic motor neurons (Liewald et al., 2008; Yang et al., 2015). The evoked EPSCs were recorded in voltage-clamped muscles with 100 ms blue light stimulation. Endogenous EPSC of *bugz-1* mutants decreased 50% in the frequency whereas the amplitudes of release events didn't altered, indicating a shrink in spontaneous release events (Figures 5A–C). The evoked response in *bugz-1* mutants also displayed a 55% decrease, suggesting a significant defect in regulated release (Figures 5D,E). The defects in both endogenous and evoked EPSCs were rescued by neuronal-expression of BuGZ-1 (Figures 5A–E). Furthermore, 1 mM acetylcholine-induced currents in body muscles didn't show any differences between wild type and *bugz-1* mutant animals (Figure 5F). These results suggest that the decrease of EPSCs at NMJs is due to decreased neurotransmitter release from motor neurons rather than altered response to neurotransmitters of body muscles.

### BuGZ-1 promotes synaptic vesicle endocytosis

**Figure 6:**
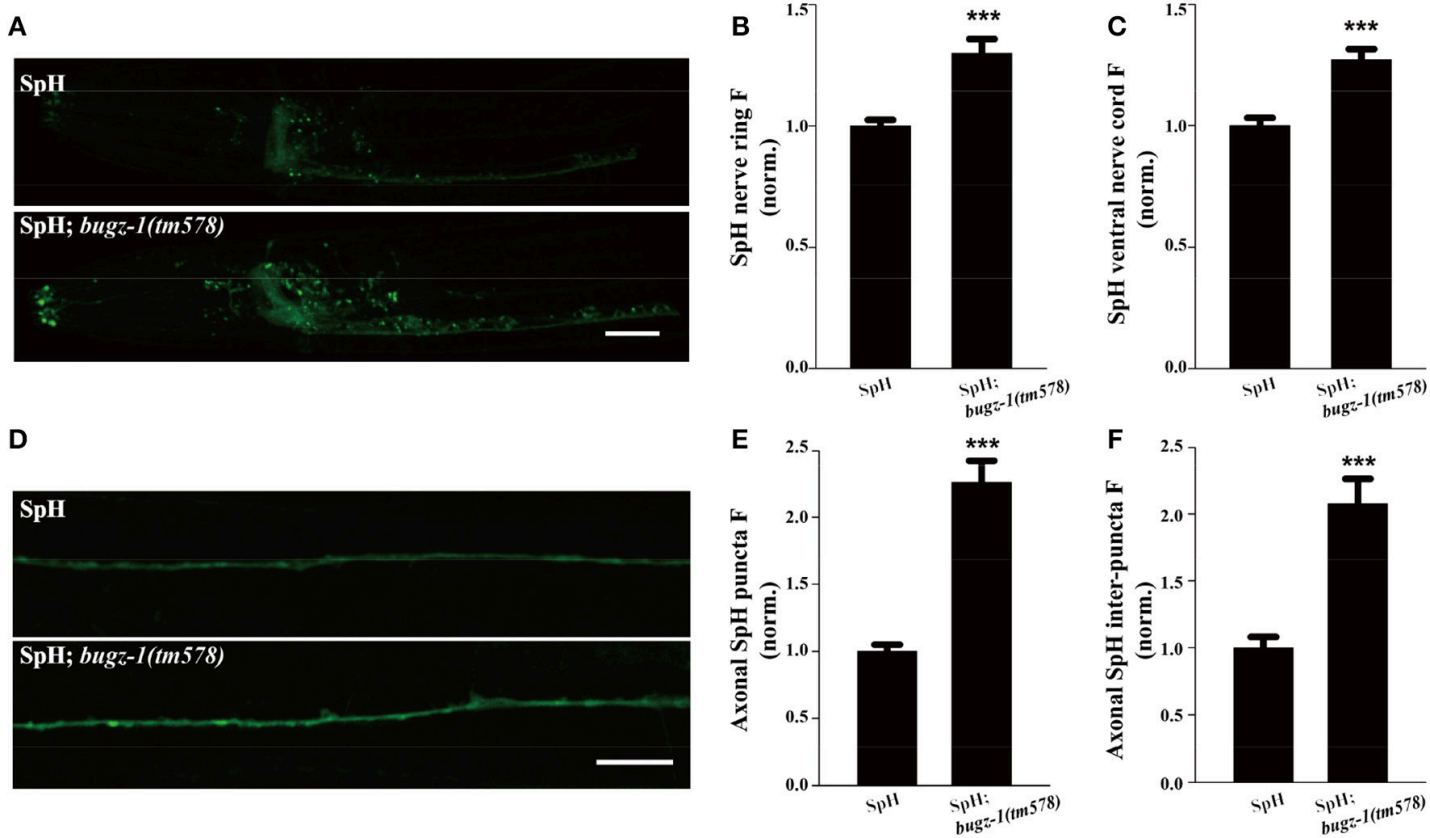
*****bugz-1(tm578)*** show synaptic vesicle endocytosis defect. (A)** Representative images of SNB-1::pHluorin (SpH) in the nerve ring and ventral nerve cord in wild type and *bugz-1(tm578)* mutant animals. **(B,C)** Quantification of fluorescence in the nerve ring **(B)** and ventral nerve cord **(C)** of *bugz-1(tm578)* mutant normalized to the wild-type control. *n* ≥ 14. **(D)** Representative images of SpH in the dorsal nerve cord in wild type and *bugz-1(tm578)* mutant animals. **(E,F)** Quantification of axonal puncta fluorescence **(E)** and inter-puncta fluorescence **(F)** of dorsal nerve cord in mutant normalized to the wild-type control. *n* ≥ 7. Values that significantly differ from controls are indicated (^***^p < 0.001 by two-tails Student's *t*-test). Error bars represent SEM. Scale bars indicate 10 μm in **(A)** and 20 μm in **(D)**.

The SpH fluorescence was largely increased when *BuGZ-1* was knocked down by RNAi treatment (1.43-fold change compared with L4440 empty vector). Significant increases of the SpH fluorescence were also observed in the nerve ring, the ventral nerve cord, as well as the axonal SpH puncta and inter-puncta of the dorsal nerve cord in *bugz-1(tm578)* mutations (Figure 6), suggesting an increase in SV exocytosis or a decrease in endocytosis in *bugz-1(tm578)* background. Given the neurotransmitter release was decreased at NMJs in *bugz-1* mutant animals (Figure 5), we propose BuGZ-1 regulates synaptic transmission by promoting SV recycling, particularly endocytosis.

### BuGZ-1 regulates the expression levels of endocytic genes

**Figure 7:**
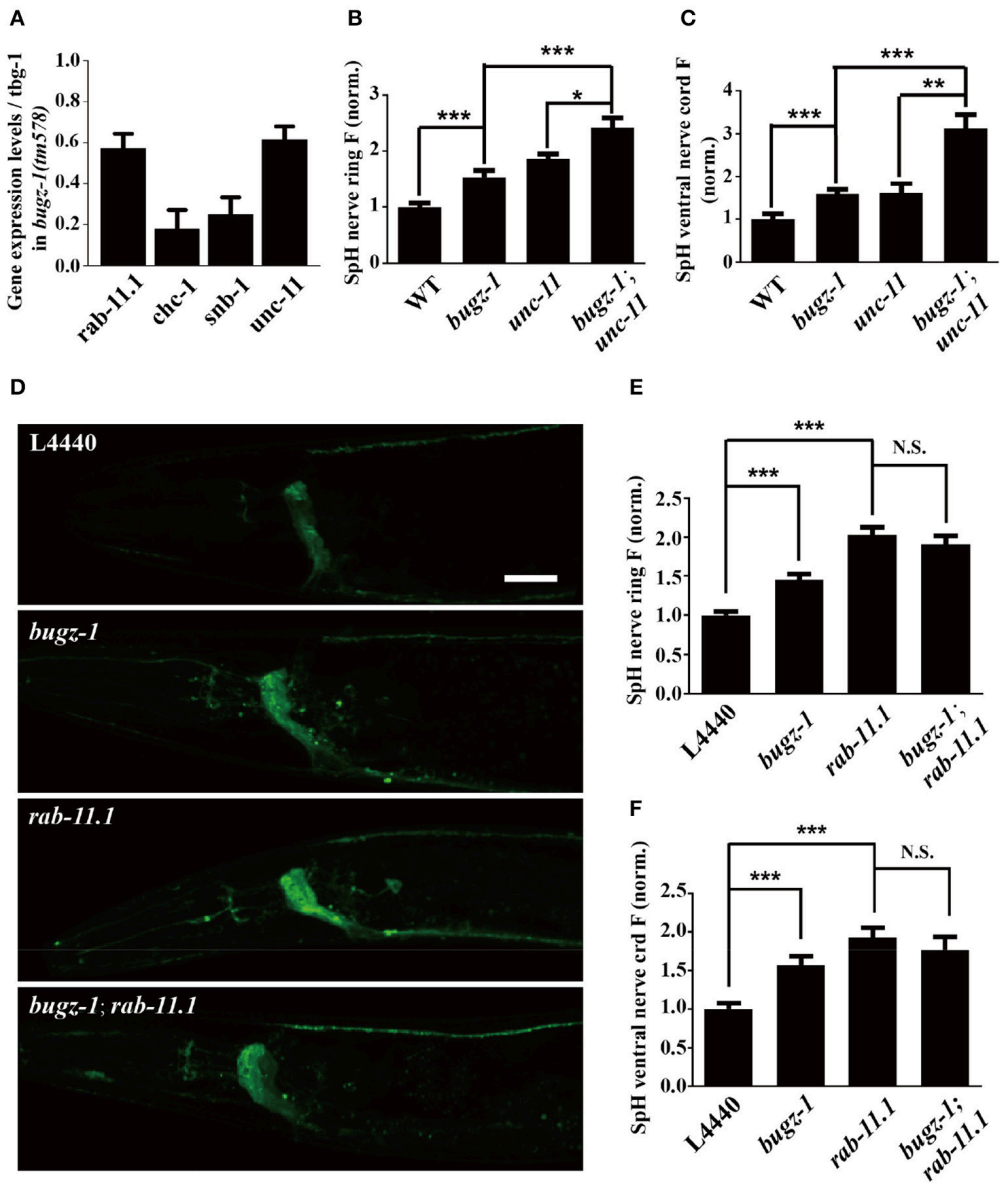
*****rab-11.1*** is a likely direct target of BuGZ-1. (A)** qRT-PCR verification of down-regulation of *rab-11.1, chc-1, snb-1*, and *unc-11* transcript levels in *bugz-1(tm578)* mutant relative to wild type. **(B,C)** Quantification of SpH fluorescence in the nerve ring **(B)** and ventral nerve cord **(C)** of N2, *bugz-1, unc-11*, and *bugz-1;unc-11* worms. *n* ≥ 14. **(D)** Representative confocal images of *eri-1(mg366);lin-15b(n744);Is[snb-1::pHluorin]* worm treated with L4440, *bugz-1, rab-11.1*, and *bugz-1;rab-11.1* RNAi bacteria. Scale bar indicates 10 μm. **(E,F)** Quantification of fluorescence in the nerve ring **(E)** and ventral nerve cord **(F)** of indicated RNAi treatment which normalized to the L4440 control. *n* ≥ 14. Values that significantly differ from controls are indicated (^*^p < 0.05; ^**^p < 0.01; ^***^p < 0.001 by two-tails Student's *t*-test). N.S. indicates no significant difference between genotypes compared. Error bars represent SEM.

The nuclei expression pattern of BuGZ-1 suggested that this protein may regulate neurotransmitter release by altering the expression level of some crucial genes related to synaptic vesicle recycling. Based on this speculation, we performed a high-throughput sequencing of *C. elegans* cDNA generated by isolating total RNA (RNA-seq) from wild type and *bugz-1(tm578)* worms (Table S4). We examined expression levels of coding sequence on a genome-wide and quantified the differences of expression levels for each gene between wild type and mutants. The expression levels of known genes essential for clathrin-mediated endocytosis including *unc-11/AP*180, *chc-1*/clathrin, and *snb-1*/synaptobrevin (Sudhof, 2004; Wu et al., 2014) were significantly reduced in *bugz-1(tm578)* worms (Figure 7A). Rab11.1, a small GTPases Rab required for endocytic recycling in many eukaryotic species (Sato et al., 2008), also showed a reduced expression level in *bugz-1(tm578)* worms (Figure 7A). We asked whether BuGZ-1 and endocytic genes function sequentially in the recycling pathway or in parallel pathways. If they function in the same pathway, simultaneous knockout or knockdown would be expected to give a phenotype similar to single mutants of either gene. However, in *bugz-1(tm578);unc-11(e47)* double mutant worms, more significant increases of the SpH fluorescence were observed both in the nerve ring and the ventral nerve cord compared to either *bugz-1(tm578)* or *unc-11(e47)* single mutant worms. These strongly enhanced phenotypes in double mutants suggest that BuGZ-1 and UNC-11 mainly function in parallel pathways, though loss of BuGZ-1 may result in down-regulation of UNC-11 (Figures 7B,C). Notably, whereas *rab-11.1(RNAi)* shows similar phenotypes with increased SpH fluorescence with *bugz-1(RNAi)* worms, no synthetic effect was observed in *bugz-1(RNAi)* combined with *rab-11.1(RNAi)* depletion, suggesting that BuGZ-1 and RAB-11.1 may function sequentially in the recycling pathway (Figures 7D–F). Considering BuGZ-1 is a predicted transcription factor and has nuclei localization, we performed chromatin immunoprecipitation followed by deep sequencing (ChIP-seq) of single copy transgenic worm *BuGZ-1::gfp* at young adult stage to determine binding sites of BuGZ-1 in the genome. We found that BuGZ-1 directly binds to *rab-11.1* (Figure S3 and Table S5), indicates that BuGZ-1 may function in endocytosis by regulating RAB-11.1, which consistent with our conclusion that BuGZ-1 and RAB-11.1 may function sequentially in SV recycling pathway.

## Discussion

In this study, we performed a high-throughput and quantitative whole-genome RNAi screen in *C. elegans* using COPAS. We identified 176 genes with up-regulated SpH fluorescence and 96 genes with down-regulated SpH fluorescence, which may be essential modulators for SV cycling. The functional classes of candidate genes indicated that diverse groups of genes, such as genes related to transcription, signaling, and kinase, transporter and channels, and membrane trafficking, are involved in SV cycling, supporting the vision that SV cycling is a complex and dynamic process involving a variety of mechanisms carried out by diverse groups of molecules.

Mammalian BuGZ/ZNF207 is a spindle assembly checkpoint protein associating with spindle microtubules and regulates chromosome alignment (Jiang et al., 2014; Toledo et al., 2014). Inhibition of BuGZ results in loss of both Bub3 and its binding partner Bub1 from kinetochores, and lethal chromosome congression defects in cancer cells (Jiang et al., 2014; Toledo et al., 2014). Nevertheless, a role of spindle assembly checkpoint proteins such as BuGZ in regulation of SV cycling have not been reported. By combining molecular genetics, optogenetics, electrophysiological recording and behavioral analysis, here we demonstrate that the *C. elegans* homolog of BuGZ is also a key modulator of synaptic vesicle recycling. Whether, BuGZ and other mitosis-associated proteins in other species have a role in synaptic transmission has yet to be identified. We envision that the functional characterization of *C. elegans* BuGZ may provide very useful insights into the functions of BuGZ and other mitosis-associated proteins in general.

The genetic interactions between *bugz-1* and endocytic genes such as *unc-11* and *rab-11.1* strongly suggest that *bugz-1* functions through, or in parallel to, clathrin-mediated endocytosis and vesicle recycling pathway during synaptic transmission. BuGZs are C2H2 zinc finger proteins, which represent the second largest gene family in humans after the odorant receptor family (Tadepally et al., 2008). Most of the characterized C2H2 zinc finger genes code for transcription factors which bind DNA through their zinc finger region; others may bind RNA and protein motifs (Wang et al., 2006; Tadepally et al., 2008). However, their exact function is as yet unknown (Tadepally et al., 2008). Given that BuGZ-1 localizes to nuclear and down-regulates the expression level of some key endocytic proteins, it is likely that BuGZ-1 functions as a transcription factor or a posttranscriptional modulator for proteins essential for endocytosis. This transcriptional or posttranscriptional modulation may provide a fine tune mechanism for synaptic transmission.

Taken together, our study provides an example that performing a high-throughput and quantitative whole-genome RNAi screen in *C. elegans*, and identified diverse groups of molecules may be involved in SV cycling. These molecules may have a broader impact on protein and vesicle trafficking. Furthermore, we have directly correlated the role of a C2H2 zinc finger spindle assembly checkpoint protein with synaptic transmission in an *in vivo* setting.

## Materials and methods

### *C. elegans* strains

All *C. elegans* strains were maintained on NGM plates at 20°C using standard methods unless otherwise statement (Brenner, 1974). The plasmid SNB-1::pHluorin (SpH, gift from Joshua Kaplan) was injected into wild-type N2 worms and integrated into chromosome by UV/TMP method and backcrossed 6 times with N2 worms to remove background mutations. The integrated strain was crossed with a RNAi hypersensitive strain KP3948 *eri-1(mg366);lin-15b(n744)* (Sieburth et al., 2005) to make the transgenic strain *eri-1(mg366);lin-15b(n744);kanIs8[snb-1::pHluorin]* for RNAi screening.

The following strains were used in this study:

*bugz-1(tm578), Y25C1A.7(tm2889), T23F11.1(tm3480), F59E12.11(tm3828), C18B12.2(tm1690)*, RB1445 *Y71G12B.11(ok1648)*, RB861 *F41D9.3(ok695)*, RB1777 *F45E4.3(ok2285)*, VC194 *Y76A2A.2(gk107)*, CB189 *unc-32(e189)*, VH624 *rhIs13[Punc-119::GFP* + *dpy-20(*+*)]; nre-1(hd20); lin-15b(hd126)*; *zxIs6[Punc-17p::chop-2(H134R)::yfp, lin-15(*+*)]* (a gift from Dr. Mei Zhen). Single copy worm strain xtl1186 *kanIs54[bugz-1p::BUGZ-1::gfp, unc-119(*+*)] II; unc-119(ed9) III*
*kanEx378[bugz-1p::BUGZ-1::gfp*+ *rab-3p::mCherry]*
*kanEx379[bugz-1p::gfp*+ *rab-3p::mCherry]*
*bugz-1(tm578); kanEx380[lin-44p::gfp*+ *rab-3p:: BUGZ-1::mCherry]*
*bugz-1(tm578); kanEx392[lin-44p::gfp*+ *myo-3p:: BUGZ-1::mCherry]*
*bugz-1(tm578);kanEx380[lin-44p::gfp*+ *rab-3p:: BUGZ-1::mCherry]*; *zxIs6[unc-17p::chop-2(H134R)::yfp, lin-15(*+*)]*
*bugz-1(tm578);kanEx392[lin-44p::gfp*+ *myo-3p:: BUGZ-1::mCherry]*; *zxIs6[unc-17p::chop-2(H134R)::yfp, lin-15(*+*)]*.

### RNAi screen

Bacterial glycerol stocks of RNAi library was replicated onto LB-agar square plates containing 25 μg/ml carbenicillin and 15 μg/ml tetracycline using a 96-pin replicator. After overnight culture at 37°C, bacterial clones were replicated from square plates into 1.2 mL 96-well plates containing 400 μl LB liquid medium with 25 μg/ml carbenicillin in each well. The RNAi bacterial clones in 1.2 mL 96-well plates were cultured overnight at 37°C with shaking. Added IPTG to bacterial cultures to a final 1 mM concentration to induce transcription of double-stranded RNA and incubated at 37°C for 1 h with shaking. Bacterial clones were spun down and resuspended with 200 μl S-Basal buffer containing 50 μg/ml carbenicillin and 1 mM IPTG. Forty microliters concentrated bacterial suspension was added into each well of standard 96-well plates. RNAi bacteria from original 96-well RNAi library were rearranged to make each experimental 96-well plate contained two L4440 controls, one *gfp* RNAi down-regulated control and one *unc-11* RNAi up-regulated control.

Adult hermaphrodite worms were bleached using standard method. Synchronized L1s were washed and resuspended in S-Basal containing 1 mM IPTG, 50 μg/ml carbenicillin, and 0.01% Tween-20. 5–8 L1s/10 μl S-Basal for F1 generation screen or 120 L1s/10 μl for P0 generation screen. Ten microliters worm solution was added into each well of 96-well plates which pre-added 40 μl concentrated RNAi bacterial suspension. Experimental 96-well plates containing bacterial clones and L1s were incubated in humid chambers at 20°C. To increase RNAi efficiency, we used two-generation RNAi treatment for whole-genome screen: synchronized L1 worms were cultured for 6 days, and fluorescent intensity of their progeny were detected by COPAS (Han et al., 2013). The RNAi clones which knocking down reduced the progeny number to less than 20% of average progeny number of the wells in the same plate were defined as sterile or lethal. We rearranged them into 25 new 96-well plates with controls. For those clones, worms were cultured for 2.5 days and L4-young adult stage of the same generation were detected by COPAS.

### Statistical analysis the screen data of COPAS

We use the relative fluorescent signal (RFS) to represent synapto-pHluorin (SpH) signal of each worm. The green fluorescent signals (represent all the SpH fluorescent signals of the worm) divided by the EXT signals (extinction integral value, represented the worm size) would result in the normalized SpH signals. Log_2_transformation was used to transform the fluorescent signals linearly. The median of RFS in each well was used to represent the signal of each well.

$$\begin{array}{l} \text{RFS}=\text{lo}{\text{g}}_{2}\frac{Green\text{ fluorescent }signals}{EXT\;signals} \end{array}$$

Robust Z-score (rZ) was used to normalize screen data in all the plates in the whole-genome screen. Robust Z-score was similar to Z-score except that the sample median and sample median absolute deviation (MAD) were used instead of sample mean and sample standard deviation, and thus was not sensitive to outliers (Birmingham et al., 2009).

$$\begin{array}{l} \text{rZ}=\frac{X-median\left(X\right)}{MAD\left(X\right)} \end{array}$$

For variable *X, MAD* is defined as the median of the absolute deviations from the median of X.

After robust Z-score normalization, the rZ score of two repeats were averaged to represent the SpH fluorescent intensity of the corresponding genes upon RNAi treatment.

In the secondary and cytoplasmic GFP screen, student's *t*-test was used to confirm the positive hits. For each plate, after removing the low repeatability genes (fold change between the two repeats was >2), one tailed *t*-test was used to identify the experimental RNAi bacteria that were significantly different from L4440 empty control.

Those RNAi bacteria with *p* < 0.05 in secondary screen and >0.05 in the cytoplasmic GFP screen were considered as positive hits.

### Aldicarb-sensitivity assay

Aldicarb-sensitivity experiments were performed on NGM plates containing 1 mM aldicarb as previously described (Wiese et al., 2012). Prepare aldicarb plates 24 h before the assay and put them at room temperature to let the plates dry. Pick 25–35 L4 worms to a fresh seeded NGM plate 16 h before the assay. Place a small spot of OP50 in the middle of aldicarb plates and let it dry thoroughly. Young adult animals were transferred to aldicarb plates and tested for paralysis every 10 min for 2 h with a harsh touch on the head. Worms failed to response to the touch were identified as paralyzed.

### Constructs and transgenes

All expression plasmids were based on the pPD95.75 vector unless otherwise statement. The putative *bugz-1* promoter region including 5 kb upstream of the gene *bugz-1*. *bugz-1p::gfp* fusion construct was generated by PCR to amplify the *bugz-1* promoter region. The PCR product of *bugz-1* promoter was digested with XmaI and AgeI–HF restriction enzymes and ligated into the pPD95.75 vector. Based on the highly consistency of functional domains between the two isoforms of *bugz-1*, we cloned the long isoform of *bugz-1* for all the rescue studies. BuGZ-1 cDNA was cloned from a cDNA library using primers targeted to the start and stop codons of the long isoform, *bugz-1b*. To generate *bugz-1p:: gfp*, we cloned *bugz-1* cDNA into *bugzp-1::GFP* using AgeI single restriction enzyme. A 1.3 kb *rab-3* promoter was used to drive *bugz-1* expression in nervous system and a 1.2 kb *myo-3* promoter was used to drive *bugz-1* expression in body muscles. *bugz-1* cDNA was digested by AgeI single restriction enzyme and then inserted into *rab-3p::mCherry* or *myo-3p::mCherry* to generate *rab-3p::BUGZ-1::mCherry* or *myo-3p::BUGZ-1::mCherry*. GFP or mCherry was fused to the C-terminus of *bugz-1* cDNA as a reporter. Transgenic worm strains were obtained by microinjection of corresponding plasmids with *Plin-44::gfp* or *Prab-3::mCherry* as a marker. For expression analysis, constructs were injected at 80 ng/μl. For rescue assays, constructs were injected at 10 ng/μl. All the markers used were injected at 30~50 ng/μl.

### Single copy insertion

Transgenic worms were generated by injection constructs into EG4322 [ttTi5605; unc-119(ed9)] animals (Frøkjaer-Jensen et al., 2008). The standard injection mix consisted of 50 ng/μl *bugz-1::gfp*, unc-119(+) repair template, 50 ng/μl Mos1 transposase pJL43.1(Pglh-2::transposase), 5 ng/μl *myo-3p::mCherry* which as a negative marker. Injected animals were transferred to NGM plates, one worm per plate. Individual injected worms were allowed to exhaust the food source of each plate. Once starved, L1 progeny were screened for insertion events with GFP fluorescence and wild-type movement but lack of co-injection marker expression.

### Image analysis

All the images were obtained using a FV 1000 laser scanning confocal microscope (Olympus). Confocal images were captured using a 60X objective with NA 1.4 at 1x or 2x digital zoom. Worms were immobilized with 30 mM NaN_3_ (Sigma) on agarose pads. For quantitative analysis, images were acquired in young adult worms and maximum fluorescent intensity of Z-series was stacked. Background fluorescence was subtracted before analysis. *P*-values were calculated by student *t*-tests. Images were quantified and analyzed using FV10-ASW Viewer and Image J. For fluorescent analysis of SpH signal in the nerve ring and ventral nerve cord, average of stacked maximum fluorescent intensity were calculated to represent florescent intensity. For fluorescent analysis in the dorsal nerve cord, florescent intensity of puncta, and inter-puncta were measured using Igor Pro software.

### Locomotion analyses

Locomotion experiments were performed by capturing movies of animals free moving on NGM plates with fresh seeded *E. Coli* OP50 8–12 h before. Fifteen young adult animals (12–14 h after L4 stage) were transferred to each 60 mm NGM plate. Ten min after transfer, a 1 min movie of animal moving was recorded using a digital camera installed on a Zeiss dissecting microscope. Movies of locomotion behaviors were analyzed by the “Imaging the Behavior of Nematodes (iBeN)” system developed in our lab. Software of iBeN system is developed based on the computer vision library, OpenCV (Open Source Computer Vision). Locomotion rates and movement status were achieved automatically by iBeN system.

### Electrophysiology

Electrophysiology assays were performed at the neuromuscular junctions of dissected *C. elegans* as previously described (Richmond and Jorgensen, 1999; Kang et al., 2010; Yang et al., 2015). Day 2 adult worms were glued on the surface of Sylgard-coated coverslips using cyanoacrylate-based glue (Zou et al., 2017), and a dorsolateral incision was made using a sharp glass pipette to expose the body wall muscles for recording. Whole-cell recordings of ventral body wall muscles were carried out by a HEKA EPC10 amplifier using the Patchmaster software. Recording pipettes were pulled from borosilicate glass capillaries (Sutter Instruments) to a resistance of 3–4 MΩ on a P-97 micropipette puller (Sutter Instruments). The bath solution contained 145 mM NaCl, 2.5 mM KCl, 5 mM CaCl2, 1 mM MgCl2, 20 mM glucose and 10 mM HEPES (325–335 mOsm, pH 7.3). The pipette solution contained145 mM KCl, 2.5 mM KCl, 5 mM MgCl2, 0.25 mM CaCl2, 10 mM HEPES, 10 mM glucose, and 5 mM EGTA, 5 mM ATP, 0.5 mM GTP (325~335 mOsm, pH 7.2). Membrane potential was clamped at −60 mV. For acetylcholine-activated experiments, 500 μM acetylcholine was perfused to the bath solution. The *zxIs6* strain, in which light-gated cation channel channelrhodopsin-2 (ChR2)-YFP was expressed in cholinergic neurons, was used for recording evoked EPSCs (Liewald et al., 2008; Yang et al., 2015). All-*trans* retinal was added to the NGM plates at a final concentration of 2.5 μM to mediate light stimulation of ChR2. L4 animals were transferred to NGM plates containing all-*trans* retinal and the next generation of 2-day-old hermaphrodite adults were used for electrophysiological studies. Blue light stimulation were performed by LAMBDA XL (Sutter Instruments) with a GFP filter controlled by the Patchmaster software.

### RNA isolation and qPCR analysis

RNA was extracted from young adult animals using Trizol reagent (Invitrogen). Total RNA was reversed to cDNA using Reverse Transcription System A3500 (Promega). For quantitative RT-PCR, Bio-Rad CFX96 real-time PCR Amplifier was used to run the cycles. qPCR was performed in triplicate for three independent biological experiments. Relative gene expression levels were calculated by ΔCt method.

### ChIP-sequencing

ChIP assays were performed as previously described (Kudron et al., 2013; Kasper et al., 2014). Young adult worms were collected after 50 h post synchronized L1. Worms were crosslinked in 2% formaldehyde for 30 min and then quenched with 1 M Tris 7.5. Worm sample was sonicated to obtain 200–800 bp DNA fragment. 4.4 mg cell extract from the sonicated worm sample was immunoprecipitated with 7.5 μg of GoatV-αGFP antibody (gift from Kevin White). Deep sequencing was performed on the Illumina Hiseq 2500 platform for the immunoprecipitated DNA fragments and genomic DNA input control. Sequencing consortium version of *C. elegans* WS235 was used to align reads. Significant binding peaks were called with SPP and IDR algorithms (Landt et al., 2012). The closest coding gene to the peak maximum of a binding site was considered as a target of the transcription factor. The ChIP-seq raw data have been uploaded to the ENCODE wedsite for public viewing and downloading (https://www.encodeproject.org/experiments/ENCSR450GPA/).

### Statistical analysis

Data analysis was performed using Excel or Igor 5. All data were presented in mean ± SEM. Unpaired two-tailed *t*-test was used for data comparison and *P* < 0.05 were considered to be statistically significant.

## Author contributions

MH, YY, LK, and TX conceived and designed the experiments. MH, WZ, HC, YY, HZ, SL, HKC, GW, and YC performed molecular genetics, optogenetics, behavioral, and electrophysiological experiments. MH, VR, LK, and TX analyzed and interpreted results. MH, YY, and LK wrote the manuscript and modified by all the other authors.

### Conflict of interest statement

The authors declare that the research was conducted in the absence of any commercial or financial relationships that could be construed as a potential conflict of interest.
