# Supplementary material for: A Systematic RNAi Screen Reveals a Novel Role of a Spindle Assembly Checkpoint Protein BuGZ in Synaptic Transmission in C. elegans
Source: Front Mol Neurosci. 2017 May 11;10:141. doi: 10.3389/fnmol.2017.00141 (PMC5425591; doi:10.3389/fnmol.2017.00141)
Supplement: Supplementary file 1 [file DataSheet1.DOCX]

Supplementary Material

**A systematic RNAi screen reveals a novel role of a spindle assembly checkpoint protein BuGZ in synaptic transmission in *C. elegans***

Mei Han^1,2,3*^, Wenjuan Zou^1*^, Hao Chang^2,3*^, Yong Yu^2*^, Haining Zhang^2^, Shitian Li^1^, Hankui Cheng^1^, Guifeng Wei^2^, Yan Chen^2^, Valerie Reinke^3^, Tao Xu^2#^, Lijun Kang^1#^

* These authors contributed equally to this work.

Correspondence:

Lijun Kang, E-mail: [kanglijun@zju.edu.cn](mailto:kanglijun@zju.edu.cn);

Tao Xu, E-mail: [xutao@ibp.ac.cn](mailto:xutao@ibp.ac.cn)

# Supplementary Figures


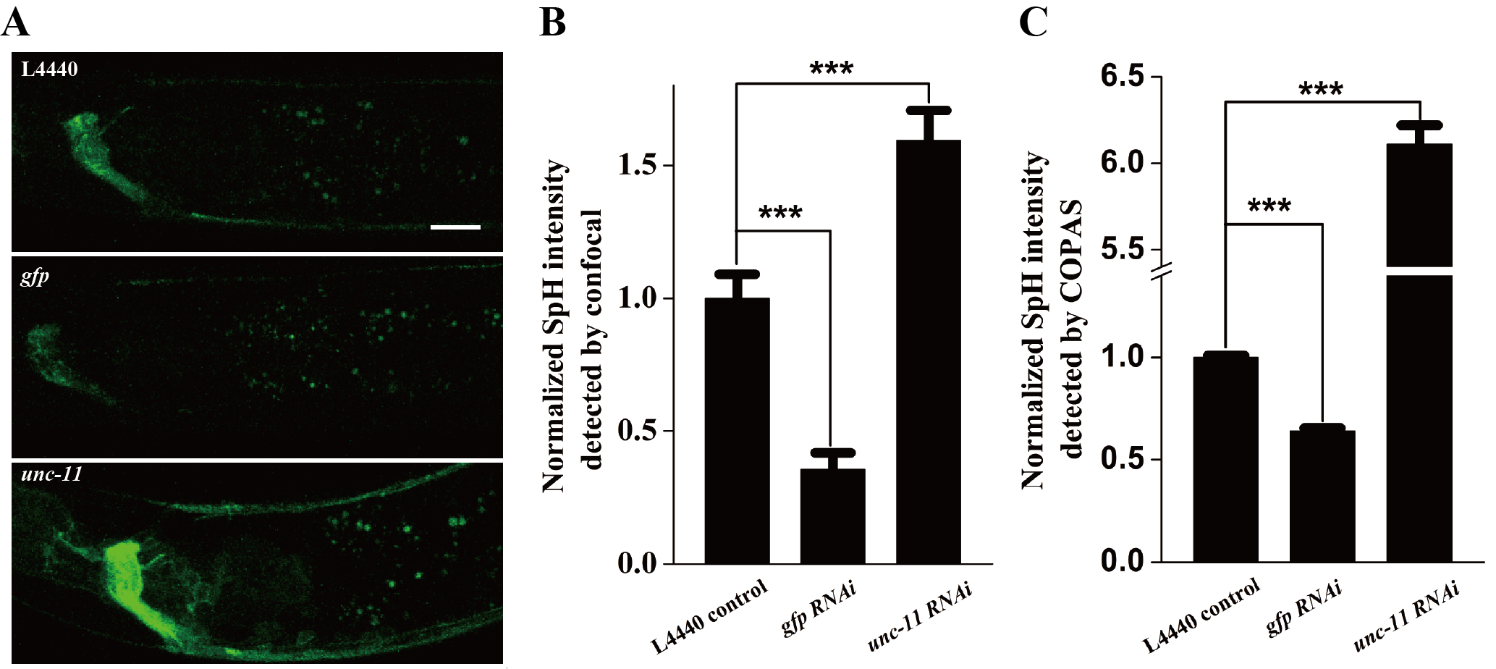


**Supplementary Figure 1.** **Detecting fluorescence of SNB-1::pHluorin (SpH) transgenic worm strain with indicated RNAi treatment via confocal and COPAS.**

(A) Representative confocal images of transgenic worm strain *eri-1(mg366); lin-15b(n744)*;*Is[SNB-1::pHluorin]* treated with L4440, *gfp*, and *unc-11* RNAi bacteria. P0 worms were treated with the corresponding RNAi bacteria and fluorescence of F1 young adult worms were imaged. (B and C) Quantification of fluorescence detected by confocal (B) and COPAS (C) in the nerve ring of worms treated with indicated RNAi bacteria. Data is normalized to L4440 control. n≥9 for confocal measurement and n≥200 for COPAS detection for each RNAi experiments. Scale bar indicates 10 µm. Error bars represent SEM. Values that significantly differ from L4440 controls are indicated (*** p < 0.001 by two-tails Student’s *t* test).

**
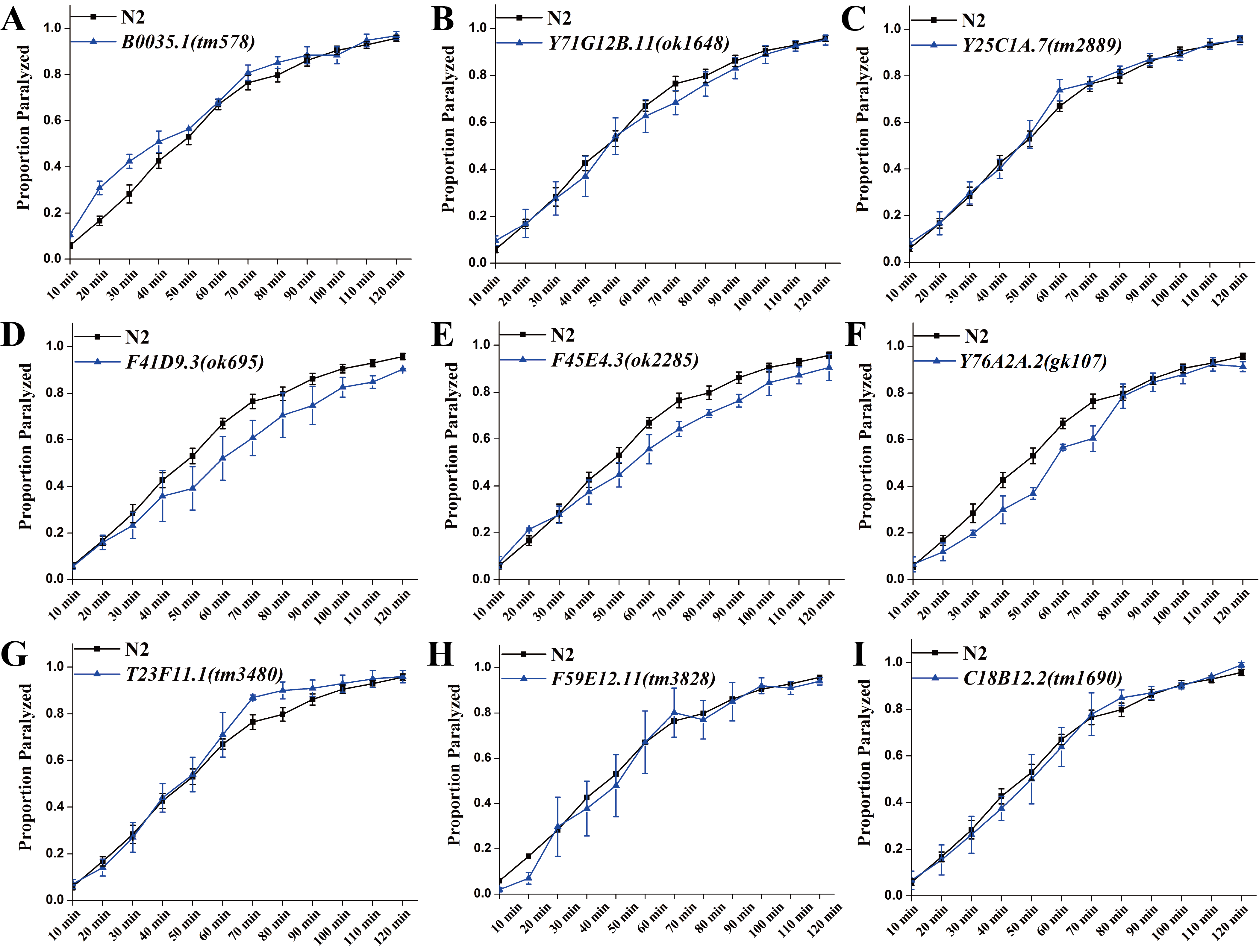
**

**Supplementary Figure 2. Mutants of candidate genes show unaltered acetylcholine receptor activity compare to wild type.**

Postsynaptic acetylcholine receptor activity was detected by determining the proportion of paralyzed animals exposed to 0.5 mM levamisole. (A-I) Proportion paralyzed over time from indicated mutant worms. Worm strains of *B0035.1(tm578)*, *Y71G12B.11(ok1648)*, *Y25C1A.7(tm2889)*, *F41D9.3(ok695)*, *F45E4.3(ok2285)*, *Y76A2A.2(gk107)*, *T23F11.1(tm3480)*, *F59E12.11(tm3828)*, and *C18B12.2(tm1690)* exhibited unaltered levamisole resistance compared to wild type. 25-35 young adult animals were tested for each experiment, at least three independent experiments were performed. Error bars represent SEM.


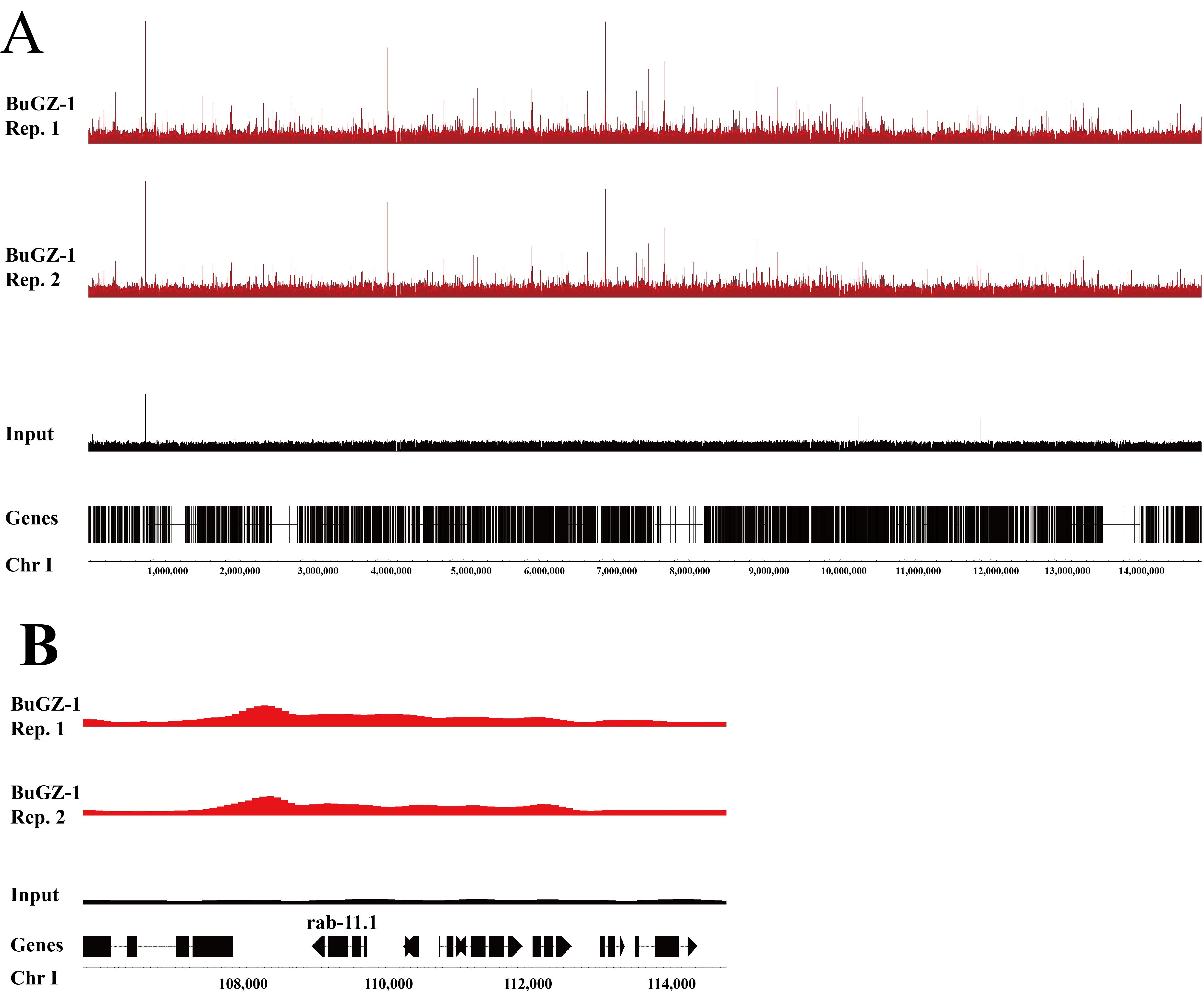


**Supplementary Figure 3. ChIP-seq binding profiles of single copy transgenic worm *BuGZ-1::GFP.***

(A) Young adult ChIP-seq binding profiles for single copy worm BuGZ-1::GFP across Chr I for two replicates and input control. Read counts for each ChIP-seq dataset are normalized by the total number of reads. (B) Binding profile of BuGZ-1::GFP at the rab-11.1 locus.

# Supplementary Tables

Table S1 Full list of the whole-genome RNAi screen in *C. elegans* to identify novel genetic modulators in SV cycling. The fluorescence of synapto-pHluorin (SpH) were captured by the Complex Object Parametric Analysis and Sorter (COPAS).

Table S2 Candidate gene for synaptic vesicle cycling revealed by multi-round RNAi screen.

Table S3 Functional classes of candidate genes.

Table S4 A high-throughput sequencing of *C. elegans* cDNA generated by isolating total RNA (RNA-seq) from wild type and *bugz-1(tm578)* worms.

Table S5 Chromatin immunoprecipitation followed by deep sequencing (ChIP-seq) of single copy transgenic worm *BuGZ-1::gfp* at young adult stage.
